# Skin Biopsy as a Diagnostic Tool for ATTRv Amyloid Neuropathy in the UK

**DOI:** 10.1111/jns.70042

**Published:** 2025-06-28

**Authors:** Luke F. O'Donnell, Victor Zhang, Roy Carganillo, Alexander M. Rossor, Matilde Laura, Mariola Skorupinska, Janet A. Gilbertson, Dorota Rowczenio, Yousuf Razvi, Julian D. Gillmore, Mary M. Reilly

**Affiliations:** ^1^ Centre for Neuromuscular Diseases, Department of Neuromuscular Diseases UCL Queen Square Institute of Neurology London UK; ^2^ National Amyloidosis Centre University College London London UK

**Keywords:** amyloid, skin biopsy, TTR

## Abstract

**Objective:**

Gene silencing therapy for ATTRv has revolutionised treatment. In minimally symptomatic, early neuropathic disease, skin biopsy can aid in the diagnosis of ATTRv‐PN, assessing both amyloid deposition and IENFD. Our aim was to study the value of performing skin biopsies in the diagnosis of ATTRv‐PN in UK patients and to assess the influence of this on accessing gene silencing treatment.

**Methods:**

Seventy‐three patients had skin biopsies performed between July 2021 and October 2023. These were stained for amyloid, typed by immunohistochemistry, and analysed for IENFD.

**Results:**

The Thr60Ala (30%), Val122Ile (23%) and Val30Met (22%) variants represented the largest number of cases. Normal/equivocal neurophysiology was demonstrated in 78% of cases. 40% of patients had abnormal IENFD, 33% had positive amyloid and 16% had both. This allowed 33% of patients to start gene silencing therapy, 75% of whom had a preceding amyloid cardiomyopathy diagnosed.

**Conclusions:**

Skin biopsy is a useful, minimally invasive method for diagnosing ATTRv‐PN. It allowed a substantial number of patients to commence gene silencing treatment. As Thr60Ala and Val122Ile are the commonest TTR variants in the UK and patients often present with cardiomyopathy, early diagnosis of ATTRv‐PN is critical for treatment decisions.

## 1 Introduction

Hereditary transthyretin amyloidosis (ATTRv) results from variants in the TTR gene [1]. The Val30Met variant is the most frequent worldwide, whereas the Thr60Ala variant is the most frequent in white Irish and British populations [1, 2]. Patients from endemic populations in Portugal and Japan produce an early‐onset (< 50 years) ATTRv‐polyneuropathy (ATTRv‐PN) phenotype, whereas Val30Met patients in northern Sweden and worldwide can produce a late‐onset (> 50 years) mixed phenotype with ATTRv‐PN and ATTRv‐cardiomyopathy (ATTRv‐CM) [1, 2]. The Thr60Ala variant produces a late‐onset mixed phenotype of ATTRv‐PN and ATTRv‐CM [1, 2]. The Val122Ile variant is usually a late‐onset ATTRv‐CM phenotype and is the second commonest cause of ATTR‐CM in the United Kingdom (UK) after wild‐type TTR (TTRwt) [3]. Furthermore, there are large African and African‐Caribbean populations in the UK, where 3%–4% are reported to carry this variant [3].

There is now targeted gene silencing therapy for ATTRv [4], which is licenced for ATTRv‐PN. It is important therefore to identify sensitive and accurate biomarkers of neuropathy enabling treatment at an earlier stage [5].

One method is skin biopsy, which can assess both amyloid deposition and intraepidermal nerve fibre density (IENFD), where reduced IENFD can signify small fibre neuropathy in the correct clinical context [6]. One study of mainly Val30Met patients identified skin amyloid deposits in 75% of early stage ATTRv‐PN patients, with 100% of symptomatic patients demonstrating reduced IENFD [6]. In this referenced study, there were no Thr60Ala patients and only 6 (3%) Val122Ile patients [6]. Similar studies looking at skin amyloid deposition in ATTRv‐PN patients have included few patients with the Thr60Ala and Val122Ile variants (range 0–2 patients) [7, 8, 9, 10, 11].

This study is the first to assess ATTRv skin biopsy samples in a UK cohort, where there is a larger cohort of Thr60Ala and Val122Ile patients. Our aim was to assess if performing skin biopsy samples in this population aided in diagnosing early ATTRv‐PN, enabling earlier gene silencing therapy.

## 2 Methods

### 2.1 ATTRv Patient Cohort

Seventy‐three ATTRv patients had a punch skin biopsy performed at the National Hospital for Neurology and Neurosurgery (NHNN), Queen Square, London, UK between July 2021 and October 2023. One patient had a TTR variant of uncertain significance (VUS) whereas the remaining 72 patients had a confirmed pathogenic variant in the TTR gene (Table 1). Out of these, 36 (50%) had ATTRv‐CM without a definite neuropathy, 32 (44%) were carriers of a pathogenic TTR variant, 3 (4%) had received a domino liver transplant with no evidence of systemic amyloidosis, and 1 patient had previously received a clinical diagnosis of ATTRv‐PN.

**TABLE 1:** Demographic and genotypic characteristics of 73 ATTRv patients.

Characteristic	ATTRv patients
Age [y], mean ± SD	59 ± 14.2
Sex female/male [*n*]	38/35
Genotypes [*n*]	Thr60Ala [22]
	Val122Ile [17]
	Val30Met [16]
	Ile68Leu [3]
	Ser77Tyr [2]
	Ile107Val [2]
	Glu42Asp [1]
	Phe33Val [1]
	Gly42Ala [1]
	Gly47Glu [1]
	His51Asn^a^ [1]
	Ala39Asp [1]
	Ile84Ser [1]
	Tyr78Phe [1]
	Tyr114Cys [1]
	Gly47Arg [1]
	Glu54Gly [1]

Abbreviations: ATTRv, hereditary transthyretin amyloidosis; SD, standard deviation.

^a^ Variant of uncertain significance (VUS).

The clinical symptoms used to diagnose neuropathy included symptoms consistent with small and/or large fibre dysfunction such as dysesthesia and paresthesia, neuropathic pain, numbness and motor weakness in distal lower extremities, respectively. Clinical signs on examination included loss of nociception and thermal sensation, impaired vibration and proprioception, muscle wasting and motor weakness, and loss of deep tendon reflexes. Both clinical symptoms and signs of a peripheral neuropathy not attributable to another cause had to be present to give a diagnosis of a definite neuropathy. The diagnosis was made by one of two consultant neurologists (MMR or AMR) and took into account the age of the patient and comorbidities such as spinal stenosis or lumbar spinal degenerative disease when defining signs as abnormal.

Skin biopsy was done in patients with (i) symptoms or signs of neuropathy on clinical examination but with normal neurophysiology; (ii) symptoms or signs of neuropathy with equivocal neurophysiology; (iii) clinically definite neuropathy with abnormal neurophysiology where there was diagnostic uncertainty as to the cause of the neuropathy, for example, co‐existent diabetes mellitus (DM) or (iv) clinically definite neuropathy where tissue confirmation of amyloid was required (Table 2). Patients who had a definite neuropathy clinically (from examination, neurophysiology, autonomic testing) and a diagnosis of ATTRv‐CM with a 3,3‐diphosphono‐1,2‐propanodicarboxylic acid (DPD) scan grade 2 or higher and no clonal plasma cell disorder were diagnosed as having a definite ATTRv‐PN not requiring a skin biopsy [2].

**TABLE 2:** Breakdown of clinical characteristics for the four predefined groups.

	Group			
	Symptoms or signs of neuropathy with normal neurophysiology	Symptoms or signs of neuropathy with equivocal neurophysiology	Clinically definite neuropathy with abnormal neurophysiology and diagnostic uncertainty	Clinically definite neuropathy needing amyloid tissue confirmation
Total	49	8	12	4
Mean age, years (SD)	58 (14.2)	71 (13.8)	61 (15.4)	63 (11.8)
Genotype (*n*)	T60A (17)	V122I (4)	V122I (5)	T60A (1)
	V30M (12)	T60A (2)	T60A (3)	V30M (1)
	V122I (11)	V30M (1)	V30M (2)	Y78F (1)
	I107V (2)	G42A (1)	H51N (1)	S77Y (1)
	A39D (1)		I68L (1)	
	E42D (1)			
	E54G (1)			
	F33V (1)			
	I68L (1)			
	I84S (1)			
	Y114C (1)			
DPD grade (*n*)	0 (22)	0 (1)	0 (5)	0 (2)
	1 (4)	1 (1)	1 (2)	1 (1)
	2 (18)	2 (6)	2 (3)	2 (1)
	3 (5)		3 (1)	
			ND (1)	
Skin biopsy results	IENFD abnormal (19)	IENFD abnormal (0)	IENFD abnormal (5)	IENFD abnormal (2)
	Amyloid+ (13)	Amyloid+ (3)	Amyloid+ (3)	Amyloid+ (3)
	Both IENFD abnormal and amyloid+ (8)	Both IENFD abnormal and amyloid+ (0)	Both IENFD abnormal and amyloid+ (1)	Both IENFD abnormal and amyloid+ (2)
Access to gene silencing therapy	17	3	3	1

Abbreviations: DPD, 3,3‐diphosphono‐1,2‐propanodicarboxylic acid scan; *n*, number of patients; ND, not done.

### 2.2 Neurophysiology

Nerve conduction studies (NCS) were performed on all patients and consisted of both large and small fibre studies. Large fibre studies consisted of both upper and lower limb sensory nerve action potentials (SNAPs) and compound muscle action potentials (CMAPs). Small fibre studies consisted of sympathetic skin responses, cutaneous silent periods and thermal thresholds.

### 2.3 Skin Biopsy Procedure

Two 3‐mm punch skin biopsies were performed 10 cm above the lateral malleolus under aseptic technique according to published criteria [12]. The samples were placed in two separate tubes, each containing 2% periodate‐lysine‐paraformaldehyde (PLP) fixative. Samples were stained for amyloid with Congo red and confirmed with apple‐green birefringence under high‐intensity cross‐polarised light, and if positive, typed by immunohistochemistry [13]. IENFD measurement was done using skin immunohistochemistry as per published guidelines [12].

## 3 Results

### 3.1 Patients and Clinical Data

**FIGURE 1:**
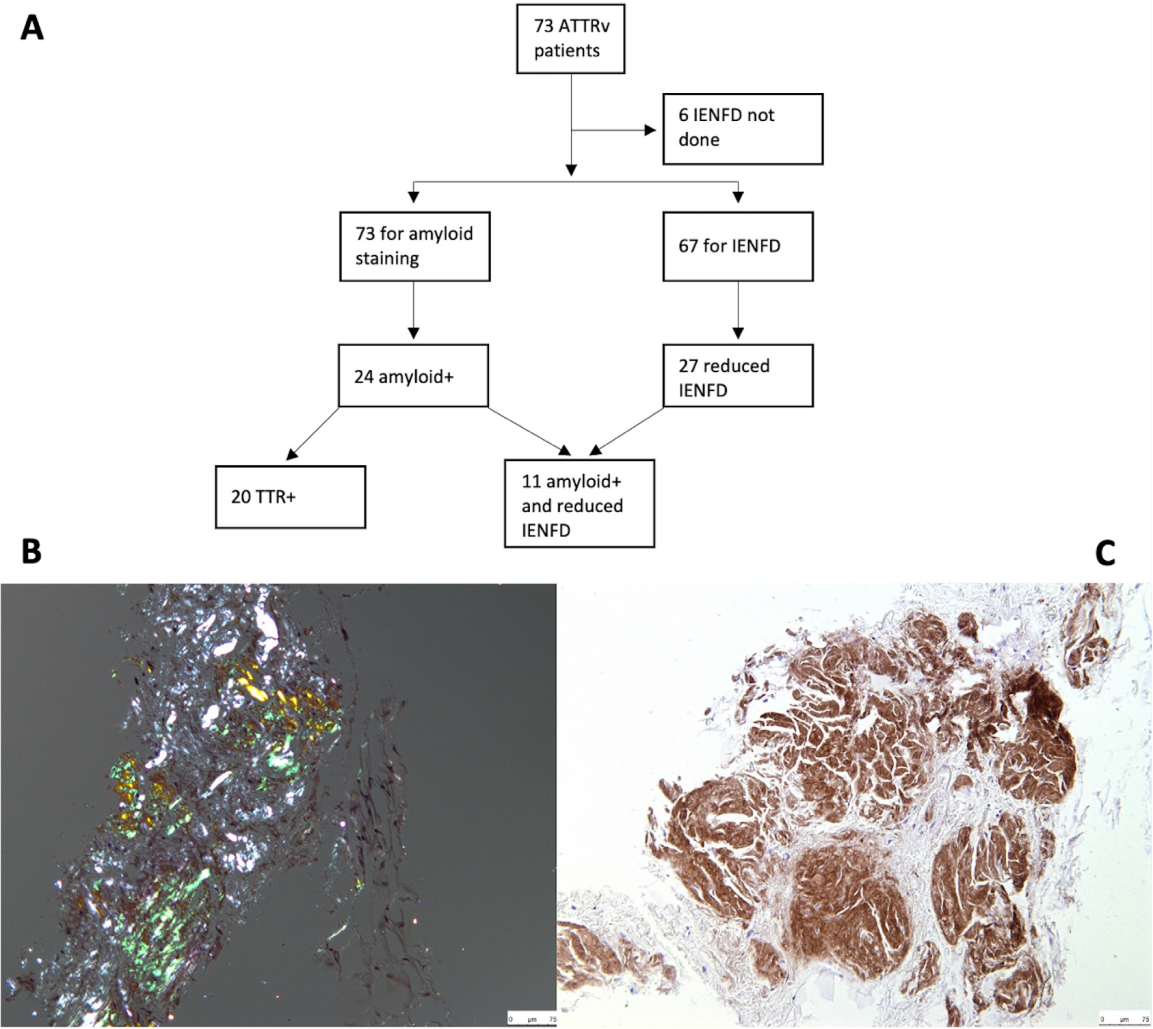
Flowchart of skin biopsy process and results of skin biopsy staining. (A) Flowchart outlining the number of patients who had amyloid staining and analysis of IENFD. (B) Skin biopsy sample with Congo red staining, viewed under cross‐polarised light, demonstrating apple‐green birefringence confirming amyloid. (C) Skin biopsy sample with amyloid stained specifically with antibodies against human transthyretin amyloid (ATTR). ATTR is stained brown. ATTR, transthyretin amyloid; IENFD, intraepidermal nerve fibre density.

Eighty‐one skin biopsies were performed on 73 patients (8 patients had a repeat biopsy at least 3 months apart; flowchart Figure 1A). The mean age of patients was 59 ± 14.2 years (range 20–86 years, Table 1). Multiple TTR variants were present, with the largest represented by the Thr60Ala variant (30%), followed by the Val122Ile (23%) and Val30Met (22%) variants. Large fibre studies were normal or equivocal in 62 (85%) of patients. Small fibre studies were completed in 64 patients and were normal or equivocal in 50 (78%). Overall, large fibre and/or small fibre studies were abnormal in 25 (34%) of patients, with large fibre studies demonstrating a length‐dependent sensory or sensorimotor axonal neuropathy. In these cases, skin biopsy was performed where there was diagnostic uncertainty as to the cause of the neuropathy (e.g., co‐existent DM) or in patients with a neuropathy clinically, and where tissue confirmation of amyloid was required. The final decision as to whether a study was abnormal was made by one of two consultant neurologists (MMR or AMR) taking into account the age of the patient, the ability of the patient to do some small fibre studies which are subjective, for example, thermal thresholds as well as the actual neurophysiology report. Many of the TTR V122I patients were of an older age and may have concomitant lumbar canal spinal stenosis.

### 3.2 Skin Biopsy Data

All 73 patients had their skin biopsy sample tested for amyloid (Figure 1A). Positive amyloid deposition was detected in 24 (33%) patients (Figure 1A,B). Out of the 24, 20 (83%) were confirmed as TTR amyloid (Figure 1A,C) and 4 (17%) could not be typed due to small sample size. 5 (21%) of the amyloid positive cases were Thr60Ala (mean age 66 ± 15 years), 2 (8%) Val122Ile (mean age 75 ± 17 years), 6 (25%) Val30Met (mean age 55 ± 15 years) and the remainder other TTR variants (Table 1). Out of the eight repeat skin biopsy samples, 2 (25%) subsequently stained positive for amyloid. IENFD was performed on 67 patients (Figure 1) and was abnormal in 27 (40%). Eleven (16%) samples were positive for amyloid and had a reduced IENFD.

The biopsy results contributed to the diagnosis of ATTRv amyloid neuropathy for each of the four predefined groups as detailed in Table 2 below.

Forty‐nine patients had symptoms or signs of neuropathy clinically but with normal neurophysiology and would be classified on the standardised polyneuropathy disability (PND) scoring system as PND‐1A. This by itself would not constitute a diagnosis of ATTRv amyloid neuropathy. All of these patients underwent skin biopsy analysis, with 17 (35%) of these patients gaining access to gene silencing therapy. Of these 17, 4 (24%) had an abnormal IENFD alone without amyloid positivity. Amyloid positivity was not necessary for diagnosis in these cases as (i) extensive testing revealed no other cause for the reduced IENFD, for example, diabetes mellitus, and (ii) their (DPD) scan was grade 2 or higher, and they were negative for a clonal plasma cell disorder as per current expert opinion and consensus [2]. Five patients (29%) had a positive TTR amyloid skin biopsy alone with a normal IENFD, and 8 (47%) had an abnormal IENFD and TTR amyloid positivity on biopsy. As can be seen from Table 1, the majority of these patients (53%) had absent or early stage cardiac disease with DPD scan grade 0 or 1.

Eight patients had symptoms or signs of neuropathy with equivocal neurophysiology. Equivocal neurophysiology referred to abnormalities on NCS such as sural SNAPs, which were classified as borderline abnormal for the patient's age. The largest group was represented by the V122I genotype, with an older mean age of 71 years. In this group, the majority of patients had established cardiac disease on DPD scan, and three gained access to gene silencing therapy based on positive TTR amyloid on skin biopsy alone.

Twelve patients had a clinically definite neuropathy with abnormal neurophysiology but there was diagnostic uncertainty due to coexisting conditions, leading to a skin biopsy for further analysis with three cases (25%) showing TTR positive amyloid on biopsy enabling gene silencing therapy to be started. Of these three cases, one patient had neuropathy possibly due to renal failure but with an abnormal IENFD and TTR amyloid positivity on biopsy, one patient with neuropathy and co‐existent DM had TTR amyloid positivity on biopsy and one patient with possible chemotherapy induced peripheral neuropathy had TTR amyloid positivity on biopsy. Again, the majority of these cases (58%) were DPD grade 0 or 1, reflecting either absent or minimal cardiac disease.

Four patients had a clinically definite neuropathy where tissue confirmation of amyloid was required, for example where there was no evidence of cardiac disease and DPD scan was grade 0–1. Three of these (75%) tested positive for TTR amyloid on biopsy.

Altogether, the positive amyloid skin biopsy results and/or reduced IENFD confirmed the diagnosis of ATTRv‐PN in 24 (33%) patients, enabling gene silencing therapy to be started. In the correct clinical context, both amyloid positivity and reduced IENFD in the skin biopsy samples (16% of patients) allowed a definitive diagnosis of ATTRv‐PN. The 16% of patients with decreased IENFD and positive amyloid deposition had an average age of 60 years, with 5 (45%) from the V30M cohort, 2 (18%) from the T60A cohort, and the remaining four patients had the Y78F, V122I, I84S and I107V TTR variants. In relation to those with negative results, the average age was 57 years, with the majority coming from T60A (11, 33%), V122I (10, 30%) and V30M (8, 24%) cohorts respectively.

Of those who started gene silencing therapy, 10 had amyloid positivity alone with normal IENFD. In these cases, the diagnosis of ATTRv amyloid neuropathy was made in the context of the overall clinical picture with symptoms and signs of neuropathy. In 33% of these cases, small fibre or large fibre studies were abnormal or were borderline abnormal for age. However, the overall clinical picture, with skin biopsy amyloid positivity, was consistent with ATTRv amyloid neuropathy. Four patients had reduced IENFD alone with negative amyloid staining. In these cases, the diagnosis of ATTRv amyloid neuropathy was made from (i) performing extensive investigations to rule out other causes of small fibre neuropathy, for example, DM and (ii) using other surrogate markers (e.g., DPD scanning) as confirmation of TTR amyloid [2].

The majority (13/24, 54%) of patients who started gene silencing therapy had minimal symptoms and/or signs of neuropathy clinically. Importantly, 18 (75%) of these patients had a preceding ATTRv‐CM. The Thr60Ala and V122Ile variants made up 9 (50%) of these ATTRv‐CM cases with a combined mean age of 70 ± 14.9 years. Of the six without a ATTRv‐CM, all of these were Val30Met patients with a mean age of 50 ± 15 years.

Fifty‐nine patients had small fibre neurophysiological studies performed and skin biopsies for measurement of IENFD. Twelve of these (20%) had abnormal neurophysiological small fibre studies, and out of these, 5 (42%) had a reduced IENFD. The remainder (47% or 80%) had normal/equivocal neurophysiological small fibre studies, and out of these, 18 (38%) had a reduced IENFD.

## 4 Discussion

Performing skin biopsy in our cohort allowed 33% of patients to start gene silencing therapy. 54% of these had minimal symptoms and/or signs of neuropathy clinically, and crucially, 75% had preceding ATTRv‐CM. The average survival of patients with ATTRv‐CM from diagnosis without treatment is 4–5 years [14]. The mortality is even higher in those with Val122Ile ATTRv‐CM [3, 15]. Of those enabled to start gene silencing therapy by a positive skin biopsy, 3 (13%) had the Val122Ile variant, all with cardiomyopathy, and 6 (25%) had the Thr60Ala variant, also all with cardiomyopathy.

There are a number of limitations with this study. We did not assess skin biopsy amyloid positivity and IENFD in healthy controls, disease controls or asymptomatic ATTRv patients. However, other studies have addressed this, demonstrating the absence of amyloid deposits in healthy controls and other non‐amyloid diseases like DM and higher amyloid positivity in ATTRv‐PN cases versus non‐ATTRv‐PN cases [6, 9]. The sensitivity and specificity of skin biopsy for detecting amyloid deposits thus remain high [6, 9]. Unlike other studies [6], we did not perform skin biopsies at a proximal site (thigh), which, if performed, may have increased our diagnostic yield [6]. However, with the length‐dependent nature of ATTRv‐PN, the ankle remains the single site with the highest yield of amyloid positivity [6]. Also, widely used normative reference ranges for IENFD do not incorporate data from healthy controls of Black African ethnicity [12], which may result in small fibre neuropathy being underdiagnosed in this subset. There is also variability in the sensitivity of Congo Red staining for amyloid deposits.

In conclusion, skin biopsy is a useful and minimally invasive method for detecting amyloid deposits and early small fibre loss. It has allowed a significant proportion of our patients to access gene silencing therapy early, the majority of whom had cardiomyopathy. Currently, we recommend considering skin biopsy for diagnosing ATTRv‐PN in the correct clinical context.

## Conflicts of Interest

The authors declare no conflicts of interest.

## Data Availability

The data that support the findings of this study are available from the corresponding author upon reasonable request.
